# Comparative risk of mortality in new users of prescription opioids for noncancer pain: results from the International Pharmacosurveillance Study

**DOI:** 10.1097/j.pain.0000000000003446

**Published:** 2024-10-29

**Authors:** Meghna Jani, Nadyne Girard, David W. Bates, David L. Buckeridge, William G. Dixon, Robyn Tamblyn

**Affiliations:** a Centre for Epidemiology Versus Arthritis, Centre for Musculoskeletal Research. The University of Manchester, UK; b Department of Rheumatology, Salford Royal Foundation Trust, Salford, United Kingdom; c NIHR Manchester Biomedical Research Centre, Manchester University NHS Foundation Trust, Manchester Academic Health Science Centre, Manchester, United Kingdom; d Department of Epidemiology, Biostatistics, and Occupational Health, University of McGill, Montreal, QC, Canada; e Clinical and Health Informatics Research Group, McGill University, Montreal, QC, Canada; f Harvard Medical School, Boston, MA, United States; g Division of General Internal Medicine, Brigham and Women's Hospital, Boston, MA, United States; h Department of Health Policy and Management, Harvard T.H. Chan School of Public Health, Boston, MA, United States

**Keywords:** Opioids, Pharmacoepidemiology, Mortality, Opioid harms, Deaths, Electronic health records

## Abstract

Although opioids continue to be used internationally for noncancer pain, evidence to date on the comparative safety of different opioids is sparse and conflicting. The aim of this study was to examine the comparative risk of all-cause mortality in patients newly initiated on opioids for noncancer pain, across 3 jurisdictions in the United Kingdom (UK), United States, and Canada. A multicentre retrospective, population-based cohort study was conducted. Data sources included UK national primary care electronic health records (Clinical Practice Research Datalink), The Partners HealthCare Research Patient Data in Boston (US), and The Montreal Population Health Record data (Canada). New users of opioids aged ≥18 years without cancer were included. Patients with a diagnosis of a pain condition and with known back pain were analysed separately. Fully adjusted hazard ratios (HRs) were calculated using Cox-proportional models and adjusted for confounders. In total, 1,066,216 patients were included (UK: n = 993,294; Boston, US: n = 43,243; Montreal, Canada: n = 26,116). Compared with codeine, patients using morphine had a significantly higher adjusted risk in the UK {HR: 12.58 [95% confidence interval (CI), 11.87-13.32]}, US (HR: 8.62 [95% CI, 3.34-22.27]), and Canadian cohorts (HR: 6.69; [95% CI, 1.35-32.22]). In addition, other factors associated with higher mortality were being on combination opioids, fentanyl, buprenorphine, and oxycodone. Compared with those on <50 morphine milligram equivalents/day, patients on higher-doses experience an incremental increase in risk. In new users of opioids, compared with codeine, strong opioids, including morphine, fentanyl, buprenorphine, oxycodone, and combination opioids, and those on ≥50 morphine milligram equivalent/day were associated with a higher subsequent risk of all-cause mortality.

## Abstract 2

Supplemental Digital Content is Available in the Text.

An international retrospective observational study evaluating the comparative safety of opioids and the effect of dose on mortality risk after adjustment of confounders.

## 1. Introduction

Over the past 2 decades, there has been a considerable surge in opioid prescriptions for noncancer pain, garnering international attention in various countries, including the United States, Canada, and the United Kingdom.^5,9,11,12^ The alarming rise in opioid-related deaths, attributed to both prescription and illicit opioids, has given rise to a major public health crisis in North America, with emerging concerns spreading across Europe. In the United States alone, deaths resulting from opioid overdoses have exhibited a steady increase, soaring from less than 20,000 in 2005 to nearly 80,500 in 2021.^16^ Similarly in Canada and the United Kingdom, opioid-related fatalities have continued to climb with approximately half of all drug-poisoning deaths in England involving an opioid in 2021.^18^ Notably, a substantial portion of individuals who use heroin initiate their drug use with prescription opioids.^3^

Despite growing awareness of the risks, opioids are still prescribed for noncancer pain, particularly in cases where therapeutic pain relief options are limited or access to nonpharmacological treatments is restricted. Musculoskeletal problems are one of the most common indications for opioid prescribing for noncancer pain.^15,20^ In patients with rheumatic and musculoskeletal diseases, there is a high frequency of patients initiating opioids who transition to long-term opioid use regardless how this is defined.^10^ Although much attention has been directed toward opioid-related deaths associated with prescription use, it is essential to recognize that the pharmacology and mechanistic properties of opioids vary, resulting in a differential risk on safety outcomes and the varied potency of opioids drugs, some opioids such as tramadol have additional serotonergic actions, whereas others such as buprenorphine are partial opioid agonists at the mu-opioid receptor. Previous findings indicate that stronger potency opioids may be linked to a higher risk of adverse events, yet the extent to which these differences are influenced by the baseline risk of the population being studied remains uncertain.

The international pharmacosurveillance study has been successful in providing timely information about drug safety and new opportunities to estimate potential sources of bias previously.^26^ We have previously published on the patient characteristics and prescribing practices across these jurisdictions previously. By evaluating mortality risk across 3 countries, we explore differences in prescribing patterns internationally, while increasing the number of outcomes for greater precision and generalizability of the results. The aim of the study was to examine the comparative risk of all-cause mortality in patients newly initiated on opioids for noncancer pain, across 3 jurisdictions in the United Kingdom, United States, and Canada.

## 2. Methods

### 2.1. Study design and population

We conducted a retrospective cohort study design to compare the mortality risk associated with patients prescribed different types of opioids. We assembled new user cohorts in the 3 jurisdictions from January 1, 2006, to January 1, 2016. We defined the index date as the date of the first opioid prescription and excluded patients who were ≥ 18 years and had one or more prescribed or dispensed opioids. We excluded patients if (1) they had an International Classification of Diseases (ICD)-10 code (United States and Canada) or a Read Code (United Kingdom) for malignancy, except for nonmelanoma skin cancer, before the index date; (2) they had a history of opioid exposure in the 2 years before their first prescription or dispensation; or (3) they were on methadone or oral/nontopical forms of buprenorphine, as these are frequently used for opioid use disorder rather than pain. We retained patients on buprenorphine patches in the analysis because these are commonly used for analgesic purposes. For simplicity, we used the term “opioid prescription” throughout. If a patient received a cancer diagnosis during follow-up, we censored them 6 months before the recorded cancer diagnosis date. We followed patients for up to 2 years after their first index opioid prescription, until the end of their registration or participation in the drug plan, or until death, whichever came first. We chose a 2-year cutoff to limit the effects of loss to follow-up in routinely collected data sets and competing risks.

We evaluated the comparative risk of death in 3 specific subgroups in each jurisdiction: all patients prescribed an opioid without cancer, patients with a pain diagnosis in the 2 years before the first opioid prescription, and patients with back or neck pain diagnosis to better address confounding by indication. For the pain diagnosis category in each cohort, we used a validated ICD-9 and ICD-10 code list mapped to Read Codes, which included lumbar, neck, and back pain, fibromyalgia, regional pain syndromes, painful neuropathic disorders, pain disorders with psychosocial dysfunction, and unclassified chronic pain.^14^ ICD-9/10 code lists for chronic noncancer pain were mapped to Read codes as published previously.^25^ For back pain specifically, we obtained established Read Codelists from www.keele.ac.uk/mrr, which have been developed with reference to clinical practice guidelines and previous consensus exercises.^28^

### 2.2. Data sources

#### 2.2.1. United Kingdom

We used the Clinical Practice Research Datalink (CPRD) Gold, a pseudonymized electronic health record database for primary care representative of the national population, to retrieve all eligible patients. We included patients in this analysis if they had linkage to the UK Office of National Statistics, which provides information on the official date of death through death registration data.

#### 2.2.2. United States, Boston, Massachusetts

We used the Partners HealthCare Research Patient Data Registry, which provides health care for 3 of the 5 million residents in Boston and its neighboring areas. The Prescription Drug Monitoring Program database provided state-wide individual-level data on prior opioid use based on dispensing information. Research Patient Data Registry includes data from the Longitudinal Medical Record, an internally developed, web-based electronic health record used during this period by the participating primary care clinics from Brigham and Women's Hospital and Massachusetts General Hospital, the 2 founding members of Partners Healthcare in Boston. We retrieved data from the structured clinical encounter information from Research Patient Data Registry for all primary care visits. To ensure complete follow-up, we included patients if they were seen in one of 37 Brigham and Women's Hospital- or Massachusetts General Hospital–affiliated primary care or diabetes clinics.

#### 2.2.3. Canada, Quebec

We used the Montreal Population Health Record data based on a population-based random sample of 25% of the 4.1 million residents of Montreal, Quebec. We retrieved hospitalization data, including admission/discharge dates, primary and secondary diagnoses, births, and deaths, from the provincial health insurance agency that provides universal medical coverage for all Quebec residents. In this jurisdiction, the first source of health coverage is the employer, who offers private insurance. Government coverage is mandatory if the employer does not offer private insurance. As a result, more people aged 65 years and older are covered through the Régie de l'assurance maladie du Québec.^22^

### 2.3. Exposure and outcome assessment

We conducted the primary analysis using time-varying exposure to opioids. We prepared opioid exposure data using a transparent drug preparation algorithm.^13,21^ Episodes of opioid use were defined using the decisions made to prepare the data described in Supplementary figure 1 (http://links.lww.com/PAIN/C158). We created mutually exclusive categories for each drug, starting with the first opioid prescription and ending with the last day of supply. We accounted for changes in opioid drug or dose by creating a new patient interval on the date of therapy change. We assigned patients on combinations of opioids a separate category. We used the Anatomic Therapeutic Classification system to map opioid names and identification numbers to a common drug nomenclature in each country. A complete list of opioids included in each country is available in eTable1 in Supplementary 1 (http://links.lww.com/PAIN/C158). We excluded injectable drugs from the analysis. We calculated morphine milligram equivalents (MMEs)/day for each patient to allow direct comparisons of opioid potency and daily dose across different formulations. We defined MME/day by multiplying the daily dose for each prescription by the analgesic ratio of the opioid type, using the US Centers for Disease Control and Prevention specified ratios, as described previously.^5,12^ All-cause mortality was the primary outcome, with death dates available from each jurisdiction's death registration data. We attributed deaths to the drug if they occurred while the patient was “on drug” rather than using an “intention-to-treat” analysis given the research question.

### 2.4. Potential confounders

We characterized the study population at the index date of the first opioid prescription, considering the following potential confounders: sociodemographic factors, comorbidities, and concurrent drug use. We grouped age into 6 categories (18-34; 35-44; 45-54; 55-64; 65-74; ≥75 years). We used the Charlson comorbidity index to measure multimorbidity and disease burden at baseline.^2^ We further evaluated specific comorbidities associated with opioid-related harms, such as depression, substance abuse, and illicit drug use as defined previously.^12^ We measured concurrent drug use associated with serious harm or mortality as time-varying covariates, including benzodiazepines, antidepressants, antipsychotics, and gabapentinoids.^8,17,19,24^ We used the Anatomic Therapeutic Classification nomenclature to identify and map local drugs names within these classes to a common data structure and created 4 binary variables to indicate the presence or absence of an active prescription in the follow-up period.

### 2.5. Statistical analysis

We compared the baseline characteristics of each of the cohorts using descriptive statistics. First, crude rates of death are presented as total events per 1000 person years. We made risk comparisons between opioids with codeine as the referent using Cox-proportional hazard models with time-varying measures of exposure of opioids and concurrent drugs. Potential confounding was reduced by adjusting for age, sex, concurrent drug use, Charlson comorbidity score, and opioid-related health problems. We stratified patients in each jurisdiction in the following 3 groups for the primary analyses: all patients without cancer prescribed an opioid, with any pain problem recorded and those with a recorded lumbar, back, or neck problem, to explore the effect of confounding by indication. We first modelled the risk of death with the use of opioids by drug substance or a combination opioid category as mutually exclusive categories periods of use and nonuse. In a second model, we evaluated the effect of daily MMEs per day (MME/day), regardless of drug substance. These analyses were performed using the MME/day thresholds from previous work aligned with international clinical guidelines.^11^ As the primary aim was to evaluate the comparative risk of death between different opioids or in combination, the results presented are those of the “on-drug” model during periods of use. Additional sensitivity analyses were performed using a 6-month and a 1-year follow-up period from index date. Statistical analyses were performed using SAS version 9.4. The study was approved by the McGill Institutional Review Board, Montreal (IRB Study number A01-E03-13B); Partners Human Research Committee (Protocol 2011P000773); and Boston and the Independent Scientific Advisory Committee for CPRD (Protocol 16_278) for UK data.

## 3. Results

### 3.1. Study population and baseline characteristics

Across the 3 jurisdictions in the study, a total of 1,062,653 patients were included (UK: n = 993,294; Boston, US: n = 43,243, Montreal, Canada: n = 26,116). The age distribution of new opioid users varied across the cohorts, with the highest proportions falling between ages of 25 and 54 years in the US and UK cohorts. In the Canadian cohort, 42.8% of patients on opioids were 65 years and older, due to how insurance is covered. More details about the descriptive prescribing differences between cohorts have been published previously.^12^

Variations were observed between the cohorts in relation to baseline comorbidities. In the Canadian cohort, depression was most prevalent (20.2%). The proportion of patients with a history of substance abuse before their first opioid prescription ranged from 3.3% in the United Kingdom to 6.8% in Canada (Table 1). The proportion of a high Charlson comorbidity score was similar across the 3 cohorts ranging between 3.2% and 4.4%. Concurrent prescriptions of benzodiazepines with opioids at the date of first opioid prescription were most common in the US (18.7%), Canadian (16.7%), and UK (5.7%) cohorts. Antidepressant use varied from 11.4% (UK), 21.6% (US), and 32.3% (Canada). Gabapentinoid use ranged from 1.0% to 6.5% and antipsychotic use between 1.8% and 7.9% (United Kingdom and Canada, respectively).

**Table 1:** Baseline characteristics of people on opioids by centre.

Patient characteristic, number (%)	United Kingdom			United States (Boston)			Canada (Montréal)		
	All patients, N = 993,294	Pain diagnosis, N = 453,601	Back or neck pain diagnosis, N = 315,608	All patients, N = 43,243	Pain diagnosis, N = 16,699	Back or neck pain diagnosis, N = 8548	All patients, N = 26,116	Pain diagnosis, N = 13,363	Back or neck pain diagnosis, N = 7035
Age at first opioid prescription or dispensation, years									
Less than 34	218,578(22.0)	101,736 (22.4)	66,098 (20.9)	9944 (23.0)	3462 (20.7)	1897 (22.2)	3215 (12.3)	1141 (8.5)	615 (8.7)
Between 35 and 44	173,116 (17.4)	86,680 (19.1)	63,219 (20.0)	8902 (20.6)	3152 (18.9)	1664 (19.5)	3027 (11.6)	1274 (9.5)	769 (10.9)
Between 45 and 54	171,193 (17.2)	84,866 (18.7)	61,631 (19.5)	9083 (21.0)	3596 (21.5)	1883 (22.0)	4140 (15.9)	1963 (14.7)	1112 (15.8)
Between 55 and 64	158,935 (16.0)	72,616 (16.0)	51,281 (16.3)	8091 (18.7)	3393 (20.3)	1604 (18.8)	4317 (16.5)	2257 (16.9)	1186 (16.9)
Between 65 and 74	128,869 (13.0)	54,483 (12.0)	37,575 (11.9)	4645 (10.7)	1930 (11.6)	926 (10.8)	5578 (21.4)	3217 (24.1)	1587 (22.6)
≥75 years	142,603 (14.4)	53,220 (11.7)	35,704 (11.3)	2578 (6.0)	1166 (7.0)	574 (6.7)	5839 (22.4)	3511 (26.3)	1766 (25.1)
Sex									
Female	575,603 (57.9)	271,166 (59.8)	179,131 (56.8)	29,194 (67.5)	11,345 (67.9)	5701 (66.7)	17,924 (68.6)	9802 (73.4)	4976 (70.7)
Male	417,691 (42.1)	182,435 (40.2)	136,377 (43.2)	14,049 (32.5)	5354 (32.1)	2847 (33.3)	8192 (31.4)	3561 (26.6)	2059 (29.3)
Charlson comorbidity index									
Score=0	644,683 (64.9)	301,412 (66.4)	215,377 (68.3)	29,991 (69.4)	10,789 (64.6)	6064 (70.9)	14,404 (55.2)	6724 (50.3)	3706 (52.7)
Low score (1)	202,655 (20.4)	92,890 (20.5)	61,823 (19.6)	7809 (18.1)	3300 (19.8)	1491 (17.4)	7026 (26.9)	3841 (28.7)	1977 (28.1)
Medium score (2-3)	105,039 (10.6)	43,515 (9.6)	28,538 (9.0)	4060 (9.4)	1914 (11.5)	745 (8.7)	3542 (13.6)	2135 (16.0)	1052 (15.0)
High score (≥4)	40,917 (4.1)	15,784 (3.5)	9770 (3.1)	1383 (3.2)	696 (4.2)	248 (2.9)	1144 (4.4)	663 (5.0)	300 (4.3)
Comorbidity in 2 years before first opioid									
Depression	87,043 (8.8)	43,578 (9.6)	28,500 (9.0)	3731 (8.6)	2122 (12.7)	1292 (15.1)	5267 (20.2)	2715 (20.3)	1455 (20.7)
Substance abuse	33,078 (3.3)	12,723 (2.8)	8386 (2.7)	1310 (3.0)	807 (4.8)	517 (6.0)	1770 (6.8)	749 (5.6)	442 (6.3)
Anxiety/PTSD	57,909 (5.8)	30,481 (6.7)	20,231 (6.4)	758 (1.8)	410 (2.4)	156 (1.8)	8022 (30.7)	4370 (32.7)	2330 (33.1)
Mental illness	8355 (0.8)	3567 (0.8)	2311 (0.7)	1741 (4.0)	901 (5.4)	341 (4.0)	2476 (9.5)	1184 (8.9)	639 (9.1)
Concurrent drug use on the date first opioid									
Benzodiazepines	56,993 (5.7)	34,709 (7.7)	29,925 (9.5)	8096 (18.7)	4113(24.6)	2246 (26.3)	4363 (16.7)	2442 (18.3)	1181 (16.8)
Antidepressants	113,322 (11.4)	54,270 (12.0)	35,510 (11.3)	9342 (21.6)	4499 (26.9)	2119 (24.8)	8430 (32.3)	4298 (32.2)	2164 (30.8)
Antipsychotics	17,441 (1.8)	6637 (1.5)	4011 (1.3)	1754 (4.1)	898 (5.4)	427 (5.0)	2051 (7.9)	908 (6.8)	495 (7.0)
Gabapentinoids	10,398 (1.0)	5569 (1.2)	3846 (1.2)	3587 (8.3)	2298 (13.8)	1105 (12.9)	1688 (6.5)	1244 (9.3)	724 (10.3)

PTSD, post-traumatic stress disorder.

Among the new opioid users without cancer, the proportion of patients presenting with a pain problem at baseline varied across the cohorts. In the US cohort, 39% of patients (n = 16,699) reported having a pain problem, whereas in the United Kingdom, this was 46% (n = 453,601), and in Montreal, this was 51% (n = 13,363) (Table 1). Of patients with a pain problem, those patients with back pain ranged between 51% (n = 8548; Boston) and 69% (n = 315,608; United Kingdom) respectively (Table 1). The baseline characteristics of patients on index date by their opioid at initiation are presented in Supplementary Table 2 (http://links.lww.com/PAIN/C158).

### 3.2. Comparative risk of death among opioids

Compared with codeine, patients using morphine had a significantly higher adjusted risk of mortality in the UK {hazard ratio (HR): 12.58 [95% confidence interval (CI), 11.87-13.32]}, US (HR: 8.62 [95% CI, 3.34-22.27]), and Canadian cohorts (HR: 6.69; [95% CI, 1.35-32.22]) (Table 2). A higher risk of death with morphine was observed across subgroups of patients with any pain diagnosis (UK: HRs, 10.29; [95% CI, 9.09-11.65]; US: HR, 5.49, [95% CI, 1.59-19.0]) and patients with back pain (UK: HR, 9.15; [95% CI, 7.69-10.89]). A similar effect was observed in fentanyl-treated patients with a higher risk of death observed in all 3 pain groups in the United Kingdom (all patients: HR, 11.35 [95% CI, 10.69-12.04]); (any pain diagnosis: HR, 8.02 [95% CI, 7.03-9.13]; back pain: HR, 6.47; 95% CI, 5.37-7.80]) and across all patients in the Canadian cohort (HR: 5.40 [95% CI, 1.12-26.05]). There were insufficient numbers of patients in the US cohort on fentanyl (Table 3). The crude incidence rates of patients on opioids across the 3 cohorts and 3 subgroups are presented in Supplementary Table 3 (http://links.lww.com/PAIN/C158).

**Table 2:** Association between opioid use and the risk of death for patients in the United Kingdom.

	All patients		Any pain patients		Back lumbar neck pain patients	
	Hazard ratio (95% CI)	*P*	Hazard ratio (95% CI)	*P*	Hazard ratio (95% CI)	*P*
Opioid class						
Codeine (referent)			Ref		Ref	
Dihydrocodeine	0.75 (0.69-0.81)	<0.001	0.74 (0.63-0.88)	<0.001	0.74 (0.59-0.92)	0.006
Buprenorphine	5.74 (5.41-6.10)	<0.001	3.93 (3.45-4.47)	<0.001	3.49 (2.91-4.18)	<0.001
Hydrocodone			na		na	
Tramadol	1.08 (1.00-1.17)	0.06	1.08 (0.93-1.25)	0.34	0.96 (0.79-1.18)	0.71
Hydromorphone			na		na	
Morphine	12.58 (11.87-13.32)	<0.001	10.29 (9.09-11.65)	<0.001	9.15 (7.69-10.89)	<0.001
Oxycodone	3.56 (2.94-4.31)	<0.001	2.47 (1.67-3.66)	<0.001	2.61 (1.59-4.31)	<0.001
Fentanyl	11.35 (10.69-12.04)	<0.001	8.02 (7.03-9.13)	<0.001	6.47 (5.37-7.80)	<0.001
Combination opioids	7.72 (7.30-8.16)	<0.001	5.88 (5.25-6.57)	<0.001	5.51 (4.74-6.40)	<0.001
Other opioids	0.86 (0.60-1.22)	0.4	0.95 (0.49-1.83)	0.88	0.56 (0.18-1.75)	0.32
Concurrent drug use						
Antidepressant	0.98 (0.95-1.01)	0.21	0.95 (0.89-1.01)	0.1	0.98 (0.90-1.06)	0.58
Antipsychotic	2.57 (2.47-2.68)	<0.001	3.02 (2.77-3.29)	<0.001	2.99 (2.66-3.35)	<0.001
Benzodiazepines	2.23 (2.15-2.32)	<0.001	2.14 (1.99-2.30)	<0.001	2.07 (1.87-2.29)	<0.001
Gabapentinoids	0.63 (0.59-0.69)	<0.001	0.73 (0.64-0.82)	<0.001	0.73 (0.62-0.87)	<0.001
Comorbidities						
Charlson score	1.23 (1.23-1.24)	<0.001	1.27 (1.26-1.28)	<0.001	1.26 (1.25-1.28)	<0.001
Depression						
No	Ref		Ref		Ref	
Yes	1.33 (1.27-1.38)	<0.001	1.47 (1.36-1.58)	<0.001	1.43 (1.30-1.58)	<0.001
Substance abuse						
No	Ref		Ref		Ref	
Yes	2.28 (2.16-2.39)	<0.001	2.23 (2.02-2.45)	<0.001	2.48 (2.19-2.81)	<0.001
Anxiety/PTSD						
No	Ref		Ref		Ref	
Yes	0.83 (0.79-0.88)	<0.001	0.87 (0.79-0.96)	0.006	0.99 (0.88-1.12)	0.9
Mental illness						
No	Ref		Ref		Ref	
Yes	1.47 (1.37-1.59)	<0.001	1.37 (1.18-1.59)	<0.001	1.41 (1.16-1.72)	<0.001
Patient gender						
Male	Ref		Ref		Ref	
Female	0.82 (0.81-0.84)	<0.001	0.79 (0.76-0.83)	<0.001	0.76 (0.72-0.80)	<0.001
Patient age						
Less than 35	Ref		Ref		Ref	
Between 35 and 44	2.15 (1.87-2.48)	<0.001	2.55 (1.95-3.33)	<0.001	2.80 (1.99-3.93)	<0.001
Between 45 and 54	3.71 (3.25-4.22)	<0.001	4.29 (3.35-5.51)	<0.001	4.45 (3.22-6.15)	<0.001
Between 55 and 64	7.02 (6.21-7.94)	<0.001	7.74 (6.09-9.84)	<0.001	8.18 (5.99-11.17)	<0.001
Between 65 and 74	16.02 (14.23-18.03)	<0.001	20.24 (16.05-25.52)	<0.001	20.70 (15.29-28.01)	<0.001
≥75 years	85.00 (75.77-95.35)	<0.001	104.49 (83.33-131.03)	<0.001	110.48 (82.18-148.52)	<0.001

CI, confidence interval; dnc, category too small, did not converge; na, drug not available; PTSD, post-traumatic stress disorder.

**Table 3:** Association between opioid use and the risk of death for patients in the United States (Boston).

	All patients		Any pain patients		Back lumbar neck pain patients	
	Hazard ratio (95% CI)	*P*	Hazard ratio (95% CI)	*P*	Hazard ratio (95% CI)	*P*
Opioid class						
Codeine (referent)	Ref		Ref		Ref	
Dihydrocodeine	na		na		na	
Buprenorphine	na		na		na	
Hydrocodone	1.06 (0.36-3.15)	0.92	0.70 (0.16-3.15)	0.64	0.92 (0.13-6.54)	0.93
Tramadol	1.06 (0.42-2.70)	0.90	0.78 (0.23-2.60)	0.68	0.76 (0.14-4.18)	0.75
Hydromorphone	1.58 (0.48-5.19)	0.45	2.25 (0.60-8.42)	0.23	2.69 (0.45-16.25)	0.28
Morphine	8.62 (3.34-22.27)	<0.001	5.49 (1.59-19.00)	0.007	3.54 (0.49-25.74)	0.21
Oxycodone	1.43 (0.61-3.32)	0.41	1.56 (0.55-4.39)	0.40	1.89 (0.44-8.08)	0.39
Fentanyl	dnc		dnc		dnc	
Combination opioids	1.49 (0.30-7.44)	0.63	dnc		dnc	
Other opioids	dnc		dnc		dnc	
Concurrent drug use						
Antidepressant	1.35 (1.09-1.68)	0.007	0.97 (0.72-1.31)	0.84	1.07 (0.69-1.66)	0.76
Antipsychotic	2.75 (2.00-3.76)	<0.001	3.30 (2.26-4.84)	<0.001	1.99 (1.12-3.53)	0.02
Benzodiazepines	1.19 (0.94-1.50)	0.15	1.17 (0.86-1.59)	0.31	1.43 (0.95-2.15)	0.09
Gabapentinoids	2.08 (1.64-2.62)	<0.001	2.33 (1.75-3.12)	<0.001	2.29 (1.52-3.44)	<0.001
Comorbidities						
Charlson score	1.25 (1.20-1.31)	<0.001	1.19 (1.11-1.27)	<0.001	0.94 (0.81-1.10)	0.44
Depression						
No	Ref					
Yes	1.91 (1.47-2.48)	<0.001	2.29 (1.66-3.16)	<0.001	2.54 (1.63-3.94)	<0.001
Substance abuse						
No	Ref		Ref		Ref	
Yes	2.02 (1.36-3.00)	<0.001	2.15 (1.39-3.34)	<0.001	2.14 (1.24-3.69)	0.006
Anxiety/PTSD						
No	Ref		Ref		Ref	
Yes	0.98 (0.48-2.03)	0.96	0.58 (0.18-1.85)	0.35	0.74 (0.10-5.53)	0.7734
Mental illness						
No	Ref		Ref		Ref	
Yes	0.96 (0.64-1.44)	0.85	0.77 (0.43-1.36)	0.37	1.00 (0.35-2.85)	0.99
Patient gender						
Male	Ref		Ref		Ref	
Female	0.83 (0.68-1.02)	0.08	0.94 (0.70-1.25)	0.65	0.91 (0.60-1.36)	0.63
Patient age, years						
Less than 35	Ref		Ref		Ref	
Between 35 and 44	4.81 (2.25-10.25)	0.40	1.31 (0.35-4.88)	0.68	0.74 (0.12-4.46)	0.74
Between 45 and 54	7.94 (3.80-16.61)	<0.001	4.69 (1.62-13.60)	0.004	3.52 (0.99-12.53)	0.05
Between 55 and 64	20.32 (9.84-41.96)	<0.001	9.57 (3.42-26.73)	<0.001	7.22 (2.14-24.41)	0.001
Between 65 and 74	62.17 (30.38-127.25)	<0.001	19.59 (7.05-54.46)	<0.001	16.37 (4.91-54.56)	<0.001
≥75 years	4.81 (2.25-10.25)	<0.001	57.92 (21.09-159.09)	<0.001	53.61 (16.48-174.43)	<0.001

CI, confidence interval; dnc, category too small, did not converge; na, drug not available; PTSD, post-traumatic stress disorder; Ref, referent.

The results of the fully adjusted models in the UK, US, and Canadian cohorts comparing between the drugs is presented in Tables 2–4, respectively. The UK cohort represented the largest cohort, with significant differences in adjusted risk of death observed between the opioids. In addition to morphine and fentanyl, the opioid drug substances associated with a higher adjusted risk of death compared with codeine in descending order of risk were as follows: being on combination of opioids (HR, 7.72 [95% CI, 7.30-8.16]), buprenorphine (HR, 5.74 [95% CI, 5.41-6.10]), and oxycodone (HR, 3.56 [95% CI, 2.94-4.31]). A significantly higher risk was also observed across the respective opioids in patients with a specific pain diagnosis and back pain diagnosis at baseline (Table 2). The results of the sensitivity analyses using a follow-up cutoff of 6 months and 1 year following index date showed similar results (Supplementary Tables 4 and 5, http://links.lww.com/PAIN/C158). The number of patients using each opioid and median number of days of use is presented in Supplementary Tables 6 to 8 (http://links.lww.com/PAIN/C158).

**Table 4:** Association between opioid use and the risk of death for patients in the Canada (Montréal).

	All patients		Any pain patients		Back lumbar neck pain patients	
	Hazard ratio (95% CI)	*P*	Hazard ratio (95% CI)	*P*	Hazard ratio (95% CI)	*P*
Opioid class						
Codeine (referent)	Ref		Ref		Ref	
Dihydrocodeine	na		na		na	
Buprenorphine	na		na		na	
Hydrocodone	na		na		na	
Tramadol	na		na		na	
Hydromorphone	0.51 (0.05-5.67)	0.59	0.93 (0.06-14.96)	0.96	0.73 (0.05-11.75)	0.83
Morphine	6.69 (1.35-33.22)	0.02	3.82 (0.34-42.40)	0.27	2.94 (0.26-32.83)	0.38
Oxycodone	0.68 (0.06-7.56)	0.76	dnc		dnc	
Fentanyl	5.40 (1.12-26.05)	0.04	2.48 (0.22-27.48)	0.46	1.76 (0.16-19.55)	0.65
Combination opioids	dnc		dnc		dnc	
Other opioids	dnc		dnc		dnc	
Concurrent drug use						
Antidepressant	0.26 (0.20-0.35)	<0.001	0.23 (0.15-0.37)	<0.001	0.27 (0.15-0.47)	<0.001
Antipsychotic	1.12 (0.75-1.68)	0.58	0.50 (0.20-1.26)	0.14	0.74 (0.26-2.11)	0.57
Benzodiazepines	0.39 (0.28-0.55)	<0.001	0.32 (0.18-0.56)	<0.001	0.41 (0.21-0.79)	0.008
Gabapentinoids	0.49 (0.30-0.81)	0.006	0.35 (0.15-0.79)	0.01	0.41 (0.17-1.01)	0.05
Comorbidities						
Charlson score	1.26 (1.20-1.32)	<0.001	1.45 (1.36-1.54)	<0.001	1.49 (1.37-1.62)	<0.001
Depression						
No	Ref		Ref		Ref	
Yes	1.88 (1.48-2.40)	<0.001	2.22 (1.54-3.20)	<0.001	2.31 (1.45-3.68)	<0.001
Substance abuse						
No	Ref		Ref		Ref	
Yes	2.43 (1.74-3.39)	<0.001	2.38 (1.39-4.08)	0.002	2.14 (1.04-4.43)	0.04
Anxiety/PTSD						
No	Ref		Ref		Ref	
Yes	0.85 (0.68-1.08)	0.19	0.99 (0.70-1.41)	0.97	1.12 (0.72-1.75)	0.62
Mental illness						
No	Ref		Ref		Ref	
Yes	1.91 (1.40-2.59)	<0.001	1.23 (0.73-2.08)	0.44	0.89 (0.42-1.85)	0.75
Patient gender						
Male	Ref		Ref		Ref	
Female	0.87 (0.70-1.07)	0.19	1.15 (0.81-1.62)	0.44	1.07 (0.68-1.68)	0.77
Patient age, years						
Less than 35	Ref		Ref		Ref	
Between 35 and 44	2.17 (0.65-7.19)	0.21	1.86 (0.34-10.18)	0.47	1.71 (0.15-18.86)	0.66
Between 45 and 54	4.32 (1.48-12.59)	0.007	1.40 (0.27-7.26)	0.69	1.60 (0.17-15.42)	0.69
Between 55 and 64	6.62 (2.32-18.86)	<0.001	3.39 (0.76-15.19)	0.11	3.64 (0.45-29.67)	0.23
Between 65 and 74	13.17 (4.78-36.28)	<0.001	5.83 (1.38-24.56)	0.02	7.70 (1.02-57.96)	0.05
≥75 years	50.69 (18.73-137.17)	<0.001	19.72 (4.82-80.72)	<0.001	22.18 (3.04-162.04)	0.002

CI, confidence interval; dnc, category too small, did not converge; na, drug not available; PTSD, post-traumatic stress disorder; Ref, referent.

### 3.3. Effect of daily opioid dose on mortality

Compared with patients on low-dose opioids (MME/day of <50MME/day), a statistically significant higher risk of death was observed in patients on MME/day of 50 or above across both the UK and US cohorts. In the UK cohort, specifically, this effect was observed across all 3 pain subgroups (Table 5). Across all patients in the UK cohort, a higher risk of death was conferred on a daily MME/day of 50 and above (HR, 5.33 [95% CI, 5.05-5.63]), with similar HRs across the 120 to 200 MME/day and ≥200 MME/day groups (Table 5). In the US cohort, an incremental increase in risk of death was observed with higher MME/day: 50 to 120 MME/day: (HR, 1.86, [95% CI, 1.15-3.01]), 120 to 200 MME/day: (HR, 6.46, [95% CI, 2.95-14.13]); ≥200 MME/day: (HR, 11.52 [95% CI, 4.94-26.86]). In the Canadian cohort, a statistically significant higher risk of death was observed in patients on ≥200 MME/day (Table 5).

**Table 5:** Fully adjusted models evaluating association between daily morphine milligram equivalents per day and the risk of death.

Centre/Dose category	All patients		Any pain patients		Back lumbar neck pain patients	
	Hazard ratio (95% CI)	*P*	Hazard ratio (95% CI)	*P*	Hazard ratio (95% CI)	*P*
United Kingdom						
<50MME/day	Ref		Ref		Ref	
50 and <120 MME/day	5.33 (5.05-5.63)	<0.001	4.57 (4.08-5.13)	<0.001	4.25 (3.62-5.00)	<0.001
120 and <200 MME/day	4.19 (3.80-4.62)	<0.001	3.82 (3.16-4.63)	<0.001	3.74 (2.84-4.93)	<0.001
>200 MME/day	4.66 (4.12-5.28)	<0.001	4.42 (3.52-5.54)	<0.001	3.64 (2.64-5.00)	<0.001
US						
<50MME/day	Ref		Ref		Ref	
50 and <120 MME/day	1.86 (1.15-3.01)	0.01	1.75 (0.94-3.26)	0.08	3.11 (1.53-6.32)	0.002
120 and <200 MME/day	6.46 (2.95-14.13)	<0.001	6.37 (2.49-16.27)	<0.001	1.72 (0.23-12.90)	0.6
>200 MME/day	11.52 (4.94-26.86)	<0.001	2.46 (0.34-18.12)	0.38	0.00 (0.00-.)	0.98
Canada						
<50MME/day	Ref		Ref		Ref	
50 and <120 MME/day	0.87 (0.19-3.91)	0.8516	3.18 (0.53-19.11)	0.2055	2.57 (0.42-15.55)	0.3049
120 and <200 MME/day	1.65 (0.21-12.80)	0.6324	dnc		dnc	
>200 MME/day	18.16 (4.99-66.08)	<0.0001	12.76 (1.28-127.69)	0.0302	8.62 (0.79-93.84)	0.0771

CI, confidence interval; dnc, category too small, did not converge; MME, morphine milligram equivalent; na, drug not available; Ref, referent.

### 3.4. Other factors associated with an increased risk of death including concomitant medications

Age was significantly associated with higher mortality risk in the ≥35 years groups (United Kingdom and United States) and ≥45 groups (Canada). Female patients had slightly lower HRs compared with male patients (United Kingdom, all patients: HR, 0.82 [95% CI, 0.81-0.84]); however, the results were not statistically significant across other cohorts and subgroups (Tables 3 and 4). A higher burden of comorbidities measured using Charlson index was associated with a higher risk of death across all cohorts (United Kingdom: HR, 1.23 [95% CI, 1.23-1.24]; United States: HR, 1.25 [95% CI, 1.20-1.31]; Canada: HR, 1.26 [95% CI, 1.20-1.32]). A history of depression and prior substance abuse were also associated with an increased risk of death across all cohorts and in most subgroups (Tables 2–4). In the UK cohort, the use of concomitant antipsychotics and benzodiazepine medication was associated with higher risk of death across all 3 subgroups (all patients, HR, 2.57 [95% CI, 2.47-2.68]; HR, 2.23 [95% CI, 2.15-2.32], respectively) as presented in Table 2. Use of antipsychotics and additionally gabapentinoids were associated with a higher mortality risk in the US cohort (all patients, HR, 2.75 [95% CI, 2.0-3.76]; HR, 2.08 [95% CI, 1.64-2.62] respectively) but not in the Canadian cohort.

## 4. Discussion

Opioid-prescribing preferences vary across different countries, but little is known regarding how differences in prescribing can influence the relative safety of patients. In this multicohort international study, we adopted a common protocol and harmonized data across cohorts to create equivalent measures of exposure opioid, including MME/day and potential confounders. The risk of all-cause mortality was significantly higher in patients prescribed morphine, fentanyl, buprenorphine, oxycodone, and on combination opioids compared with codeine. Higher MME/day was associated with an increased risk of death, particularly an MME of ≥50/day in both UK and US cohorts.

In the evaluation of comparative risk of mortality in opioid-treated patients, previous studies have shown conflicting results. An earlier study compared the association of tramadol with codeine dispensation on all-cause mortality using data from a large Spanish cohort, reporting a higher risk with tramadol (HR, 2.31 [95% CI, 2.08-2.56]).^27^ Two other studies^4,23^ based in the United States and Denmark, comparing tramadol with codeine showed a lower risk of all-cause mortality and a UK-based study showed no difference in patients older than 50 years with osteoarthritis.^29^ Solomon et al.^23^ conducted a retrospective cohort study using data from 31,375 Medicare patients and compared the safety profile of 5 commonly prescribed opioids in the United States at the time (1996-2005): hydrocodone, codeine, oxycodone, tramadol, and propoxyphene (the latter drug subsequently withdrawn because of safety concerns). In this propensity-matched cohort, compared with hydrocodone users, codeine and oxycodone users had a significantly higher rate ratio (RR) for all-cause mortality (RR: 2.05 [1.22-3.45]; RR: 2.43 [1.47-4.00] respectively), with no significantly higher risk observed in tramadol users (RR: 1.33 [0.76-2.44]). Another recent nationwide Danish study^4^ focused on 33,377 new opioid users with inflammatory bowel disease compared adverse outcomes of tramadol compared with what were termed “traditional opioids,” which included oxycodone, morphine, fentanyl, and hydrocodone among others. It reported a lower risk of all-cause mortality in new users of tramadol (adjusted odds ratio: 0.43 [95% CI, 0.35-0.55]) within 90 days after the initial use. The reported differences may be because of residual confounding and differences in baseline risk of death of the population evaluated.

This is the first study that we are aware of to examine comparative mortality risk in new users of opioids across several jurisdictions, using a harmonized protocol and methods. Our previous work highlighted the differences in prescribing patterns internationally, as well as differences in baseline characteristics of patients being initiated on opioids.^12^ Our findings from this work suggest an increased risk of mortality with stronger opioids after attempted adjustment for confounding, with a higher MME/day being associated with a higher mortality risk. However, a consistent trend across all opioids in the same direction for each cohort was not observed, partly due to fewer numbers of specific opioids such as dihydrocodeine and hydrocodone in some of the cohorts as a result of differences in prescribing preferences. The effect of dose and MME/day has also been less frequently evaluated on mortality in previous studies, as such information can be missing completely in some cases,^4^ or a proxy for dose such as the number of drug packages dispensed is used.^27^ The results of our work are consistent with the US Centers for Disease Control and Prevention 2022 recommendations^6^ and Canadian recommendations for noncancer pain,^1^ which highlight that opioid prescribers should carefully reassess individual benefits and risks of escalating doses beyond 50 MME/day as they are more likely to yield diminishing returns with increased risk. Both UK and US data demonstrated a higher risk beyond this threshold (Table 5), with considerably higher HRs in the US and Canadian patients on daily doses more than 120 MME/day. Interestingly, the Faculty of Pain Medicine treatment recommendations in the United Kingdom currently have a higher threshold of 120 MME/day as the threshold above which harms outweigh benefits and suggest opioid tapering.^7^ Our work informed by the UK data (Table 5) suggests a lower threshold similar to the United States and Canada.

The findings from our study are consistent with the US Food and Drug Adminstration black box warning against the coprescription of antipsychotics, benzodiazepines, and opioids due to the risk of respiratory depression. The use of concomitant antipsychotics with opioids was consistently associated with a higher risk of mortality in both the US and UK cohorts (Tables 2 and 3). A recent population-based cohort study supports these findings and also reported that sedating antipsychotics (such as quetiapine, olanzapine) were associated with higher risk of overdose when coprescribed with opioids compared with nonsedating antipsychotics.^24^ Coprescription of benzodiazepines and gabapentinoids with opioids were associated with an approximately 2-fold increased risk in the UK and US cohorts, respectively, in all pain subgroups. This is consistent with previous work that have also highlighted risk of death with these classes of drugs.^8,19^ The results for benzodiazepines and gabapentinoids were not consistently replicated across all 3 cohorts, however. One possible reason may be that the effect of these drug classes on opioids is dose dependent. It is likely that prescribed doses of these drug classes are different on a population level across countries, which we were not able to adjust for.

### 4.1. Limitations

The results need to be considered in light of the limitations of the data and methods. First, although we adjusted for baseline factors and explored analysis across 3 pain groups, confounding by indication could have affected the findings. For instance, oxycodone and morphine may be channelled to patients with specific comorbidities because of local prescribing guidelines that may explain some of the observed findings. Second, although we excluded patients with history of cancer before index date, opioids may be prescribed for a wide range of indications and may lead to protopathic bias, where an opioid may be inadvertently prescribed for an early manifestation of cancer (eg, pain) that may not have been diagnosed yet. To mitigate this, we censored 6 months before a diagnosis of cancer during follow-up. Excluding cancer patients may only deal with confounding by indication, where cancer is the indication for opioids; however, there may be other confounding by indication that has not been coded, which is itself associated with mortality. In addition, we evaluated separate patient subgroups initiating opioids with any pain diagnosis at baseline and a back pain diagnosis. The comparative safety results were consistent across these 3 subgroups, particularly in the largest UK cohort (Table 2).

Third, this study did not assess cause of death. Jurisdictions may capture this differently (eg, Coroner report/death certificate data) leading to inconsistencies, and this level of data was not available in all centres. Fourth, although the total number of patients across the study was high, the differences in prescribing patterns between centres meant that there were sometimes insufficient sample sizes in specific opioid groups that led to an inability of the models to converge.

Fifth, although exposure was captured using prescribing/dispensation information, adherence to opioids may be variable and may be administered as required leading to some exposure misclassification. However, this is likely to be to the same degree across the groups (nondifferential misclassification), which would bias toward the null and therefore would not explain the observed associations.

### 4.2. Conclusions

In this international multicohort study that included patients newly initiated on an opioid, significant differences in risk of death between opioids were observed. Compared with codeine, stronger opioids, such as morphine, fentanyl, buprenorphine, oxycodone, and combination opioids, were associated with a higher risk of death. Mortality risk was higher in patients on 50MME/day or more, in the UK and US cohorts specifically. Certain drug classes used concomitantly with opioids particularly antipsychotics (in United Kingdom/United States), benzodiazepines (United Kingdom), and gabapentinoids (United States) also conferred a higher risk of death.

## Conflict of interest statement

W.G.D. has received consultancy fees from Google unrelated to this work. D.W.B. reports grants and personal fees from EarlySense, equity from ValeraHealth, equity from Clew, equity from MDClone, personal fees and equity from AESOP, personal fees and equity from Feelbetter, equity from Guided Clinical Solutions, and grants from IBM Watson Health, unrelated to this work. The authors have no conflicts of interest to declare.

## Appendix A. Supplemental digital content

Supplemental digital content associated with this article can be found online at http://links.lww.com/PAIN/C158.
