# Supplementary material for: Comparative risk of mortality in new users of prescription opioids for noncancer pain: results from the International Pharmacosurveillance Study
Source: Pain. 2024 Oct 29;166(5):1118–27. doi: 10.1097/j.pain.0000000000003446 (PMC12004978; doi:10.1097/j.pain.0000000000003446)
Supplement: Supplementary file 1 [file jop-166-1118-s001.pdf]

Supplementary Material

Supplementary Table 1: Opioid type prescribed in each centre

Opioid	Montreal	Boston	UK
Codeine	Yes	Yes	Yes
Dihydrocodeine	No	No	Yes
Buprenorphine (patches)	No	Yes	Yes
Hydrocodone	No	Yes	No
Tramadol	No	Yes	Yes
Hydromorphone	Yes	Yes	No
Morphine	Yes	Yes	Yes
Oxycodone	Yes	Yes	Yes
Fentanyl	Yes	Yes	Yes
Other opioids	Tramadol<br>Meperidine<br>Butorphanol<br>Pentazocine<br>Buprenorphine<br>Tapentadol	Oxymorphone<br>Meperidine<br>Tapentadol<br>Butorphanol<br>Buprenorphine	Dextropropoxyphene<br>Meptazinol Pethidine<br>Dipipanone<br>Tapentadol<br>Papaveretum<br>Hydromorphone<br>Pentazocine<br>Diamorphine

Supplementary Figure 1: Decisions made for drug preparation to derive episodes of opioid use

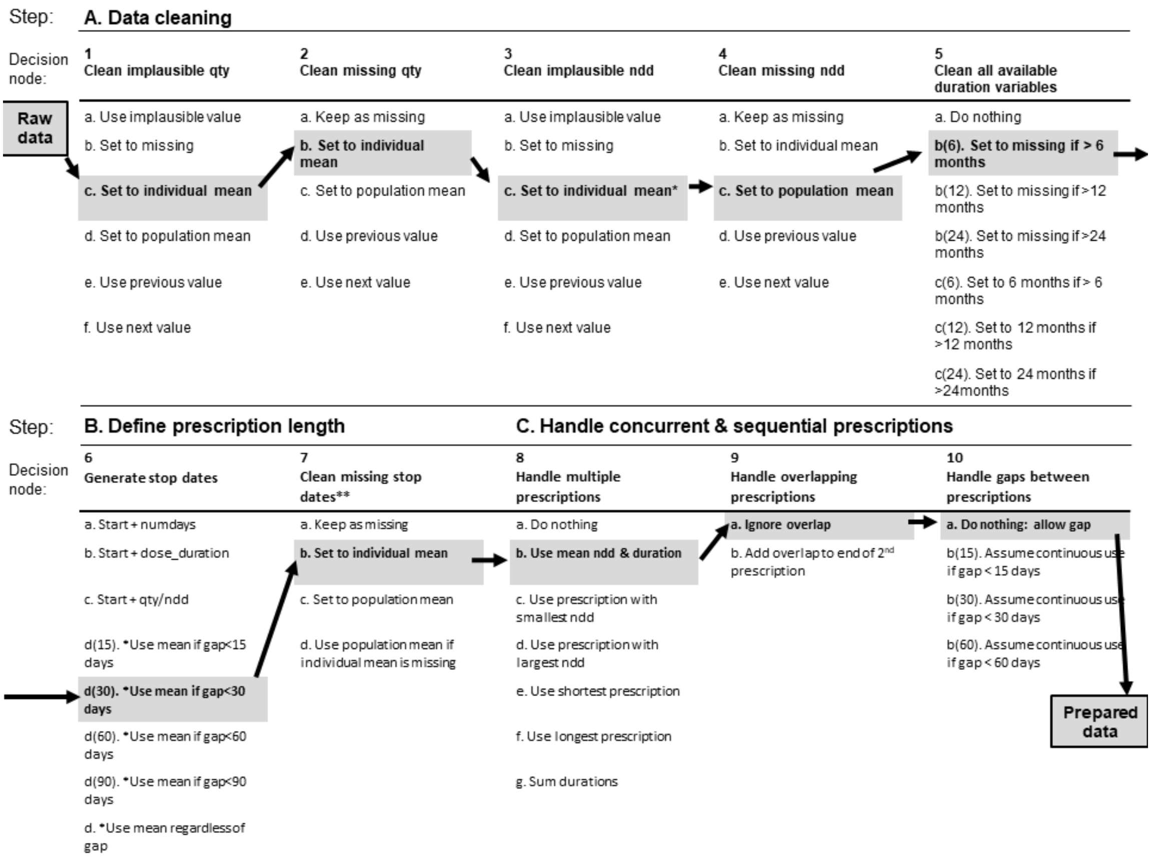

Adapted from references 13 and 21. *Decision 3c: We set to mean for individual's prescriptions for that drug; if missing set to mean for practice's prescriptions for that drug; if not available set to mean for populations' prescriptions for that drug.

Supplementary Table 2 Baseline characteristics of patients by drug at initiation by jurisdiction

United Kingdom	Codeine		Buprenorphine		Dihydrocodeine		Fentanyl		Morphine		Oxycodone		Tramadol		Other opioids		Combination opioids	
	N	%	N	%	N	%	N	%	N	%	N	%	N	%	N	%	N	%
**Patient age**																		
Less than 34 at index date	163531	23.03	91	1.93	36442	21.45	63	2.41	751	10.03	157	12.07	16035	18.52	343	9.69	1165	16.52
Between 35 and 44 at index	122841	17.3	158	3.35	30713	18.08	104	3.97	776	10.36	180	13.84	16631	19.21	446	12.6	1267	17.97
Between 45 and 54 at index	120237	16.93	304	6.45	29981	17.65	131	5	883	11.79	237	18.22	17414	20.11	642	18.14	1364	19.34
Between 55 and 64 at index	112367	15.82	421	8.93	27970	16.46	171	6.53	865	11.55	221	16.99	15109	17.45	650	18.36	1161	16.47
Between 65 and 74 at index	92200	12.98	616	13.07	22364	13.16	238	9.09	948	12.66	193	14.83	10804	12.48	629	17.77	877	12.44
More or equal 75 on index	98930	13.93	3124	66.27	22410	13.19	1912	73	3265	43.6	313	24.06	10602	12.24	830	23.45	1217	17.26
**Sex**																		
Male	296863	41.81	1196	25.37	72845	42.88	736	28.1	3143	41.97	559	42.97	37956	43.83	1274	35.99	3119	44.23
Female	413243	58.19	3518	74.63	97035	57.12	1883	71.9	4345	58.03	742	57.03	48639	56.17	2266	64.01	3932	55.77
**Charlson score at index date**																		
Score=0	466312	65.67	1189	25.22	113419	66.76	511	19.51	2582	34.48	593	45.58	54177	62.56	2163	61.1	3737	53
Low score (1)	143449	20.2	1073	22.76	34288	20.18	640	24.44	1729	23.09	302	23.21	18820	21.73	757	21.38	1597	22.65
Medium score (2-3)	73076	10.29	1468	31.14	16325	9.61	829	31.65	1764	23.56	231	17.76	9770	11.28	463	13.08	1113	15.78
High score (≥4)	27269	3.84	984	20.87	5848	3.44	639	24.4	1413	18.87	175	13.45	3828	4.42	157	4.44	604	8.57
**Depression**																		
No	648909	91.38	4193	88.95	155659	91.63	2307	88.09	6596	88.09	1127	86.63	78086	90.17	3232	91.3	6142	87.11
Yes	61197	8.62	521	11.05	14221	8.37	312	11.91	892	11.91	174	13.37	8509	9.83	308	8.7	909	12.89
**Anxiety**																		
No	668279	94.11	4518	95.84	160466	94.46	2525	96.41	7076	94.5	1224	94.08	81278	93.86	3338	94.29	6681	94.75
Yes	41827	5.89	196	4.16	9414	5.54	94	3.59	412	5.5	77	5.92	5317	6.14	202	5.71	370	5.25
**Mental illness**																		
No	704339	99.19	4619	97.98	168584	99.24	2542	97.06	7331	97.9	1289	99.08	85764	99.04	3506	99.04	6965	98.78

Yes	5767	0.81	95	2.02	1296	0.76	77	2.94	157	2.1	12	0.92	831	0.96	34	0.96	86	1.22
**Substance abuse**																		
No	687844	96.86	4552	96.56	165259	97.28	2492	95.15	6831	91.23	1164	89.47	82143	94.86	3477	98.22	6454	91.53
Yes	22262	3.14	162	3.44	4621	2.72	127	4.85	657	8.77	137	10.53	4452	5.14	63	1.78	597	8.47

**United States (Boston)**	**Codeine**		**Fentanyl**		**Hydrocodone**		**Hydromorphone**		**Morphine**		**Oxycodone**		**Tramadol**		**Other opioids**		**Combination opioids**	
	**N**	**%**	**N**	**%**	**N**	**%**	**N**	**%**	**N**	**%**	**N**	**%**	**N**	**%**	**N**	**%**	**N**	**%**
**Patient age**																		
Less than 34 at index date	690	19.06	5	7.46	878	17.78	356	20.33	32	10.36	7481	26.53	482	11.51	8	14.04	12	11.01
Between 35 and 44 at index	772	21.32	9	13.43	946	19.16	396	22.62	37	11.97	6202	21.99	521	12.44	7	12.28	12	11.01
Between 45 and 54 at index	756	20.88	16	23.88	1176	23.82	358	20.45	68	22.01	5793	20.54	883	21.08	14	24.56	19	17.43
Between 55 and 64 at index	677	18.7	12	17.91	1094	22.16	377	21.53	76	24.6	4819	17.09	996	23.78	14	24.56	26	23.85
Between 65 and 74 at index	456	12.59	10	14.93	571	11.57	195	11.14	44	14.24	2621	9.29	709	16.93	9	15.79	30	27.52
More or equal 75 on index	270	7.46	15	22.39	272	5.51	69	3.94	52	16.83	1287	4.56	598	14.28	5	8.77	10	9.17
**Sex**																		
Male	1041	28.75	22	32.84	1676	33.95	487	27.81	124	40.13	9464	33.56	1171	27.95	19	33.33	45	41.28
Female	2580	71.25	45	67.16	3261	66.05	1264	72.19	185	59.87	18739	66.44	3018	72.05	38	66.67	64	58.72
**Charlson score at index date**																		
Score=0	2600	71.8	43	64.18	3701	74.96	1002	57.22	179	57.93	19726	69.94	2641	63.05	37	64.91	62	56.88
Low score (1)	670	18.5	10	14.93	830	16.81	382	21.82	51	16.5	4989	17.69	839	20.03	12	21.05	26	23.85
Medium score (2-3)	272	7.51	10	14.93	326	6.6	249	14.22	42	13.59	2604	9.23	536	12.8	4	7.02	17	15.6
High score (≥4)	79	2.18	4	5.97	80	1.62	118	6.74	37	11.97	884	3.13	173	4.13	4	7.02	4	3.67
**Depression**																		
No	3345	92.38	52	77.61	4532	91.8	1555	88.81	256	82.85	25847	91.65	3778	90.19	46	80.7	101	92.66
Yes	276	7.62	15	22.39	405	8.2	196	11.19	53	17.15	2356	8.35	411	9.81	11	19.3	8	7.34
**Anxiety**																		
No	3553	98.12	64	95.52	4881	98.87	1701	97.14	296	95.79	27747	98.38	4086	97.54	52	91.23	105	96.33

Yes	68	1.88	3	4.48	56	1.13	50	2.86	13	4.21	456	1.62	103	2.46	5	8.77	4	3.67
**Mental illness**																		
No	3487	96.3	61	91.04	4805	97.33	1659	94.75	287	92.88	27117	96.15	3940	94.06	49	85.96	97	88.99
Yes	134	3.7	6	8.96	132	2.67	92	5.25	22	7.12	1086	3.85	249	5.94	8	14.04	12	11.01
**Substance abuse**																		
No	3552	98.09	64	95.52	4809	97.41	1696	96.86	280	90.61	27305	96.82	4067	97.09	55	96.49	105	96.33
Yes	69	1.91	3	4.48	128	2.59	55	3.14	29	9.39	898	3.18	122	2.91	2	3.51	4	3.67

**Canada (Montreal)**	**Codeine**		**Fentanyl**		**Hydromorphone**		**Morphine**		**Oxycodone**		**Other opioids**		**Combination opioids**	
	**N**	**%**	**N**	**%**	**N**	**%**	**N**	**%**	**N**	**%**	**N**	**%**	**N**	**%**
**Patient age**														
Less than 34 at index date	1936	14.17	1	0.66	506	10.07	350	12.04	346	9.34	41	12.5	35	10.51
Between 35 and 44 at index	1712	12.53	9	5.96	555	11.05	290	9.97	374	10.09	42	12.8	45	13.51
Between 45 and 54 at index	2280	16.68	10	6.62	832	16.56	381	13.1	523	14.11	62	18.9	52	15.62
Between 55 and 64 at index	2289	16.75	9	5.96	831	16.54	465	15.99	601	16.22	58	17.68	64	19.22
Between 65 and 74 at index	2781	20.35	26	17.22	1133	22.56	642	22.08	856	23.1	71	21.65	69	20.72
More or equal 75 on index	2669	19.53	96	63.58	1166	23.21	780	26.82	1006	27.15	54	16.46	68	20.42
**Sex**														
Male	4219	30.87	35	23.18	1641	32.67	915	31.46	1186	32	74	22.56	122	36.64
Female	9448	69.13	116	76.82	3382	67.33	1993	68.54	2520	68	254	77.44	211	63.36
**Charlson score at index date**														
Score=0	7789	56.99	55	36.42	2722	54.19	1554	53.44	1910	51.54	185	56.4	189	56.76
Low score (1)	3719	27.21	42	27.81	1285	25.58	771	26.51	1037	27.98	96	29.27	76	22.82
Medium score (2-3)	1653	12.09	36	23.84	770	15.33	414	14.24	584	15.76	39	11.89	46	13.81
High score (≥4)	506	3.7	18	11.92	246	4.9	169	5.81	175	4.72	8	2.44	22	6.61
**Depression**														
No	11003	80.51	112	74.17	4057	80.77	2258	77.65	2904	78.36	258	78.66	257	77.18
Yes	2664	19.49	39	25.83	966	19.23	650	22.35	802	21.64	70	21.34	76	22.82
**Anxiety**	9599	70.23	111	73.51	3627	72.21	2064	70.98	2676	72.21	218	66.46	230	69.07
No														
Yes	4068	29.77	40	26.49	1396	27.79	844	29.02	1030	27.79	110	33.54	103	30.93

**Mental illness**														
No	12340	90.29	133	88.08	4582	91.22	2612	89.82	3365	90.8	304	92.68	304	91.29
Yes	1327	9.71	18	11.92	441	8.78	296	10.18	341	9.2	24	7.32	29	8.71
**Substance abuse**														
No	12739	93.21	145	96.03	4685	93.27	2693	92.61	3474	93.74	307	93.6	303	90.99
Yes	928	6.79	6	3.97	338	6.73	215	7.39	232	6.26	21	6.4	30	9.01

Supplementary Table 3: Crude incidence rates of patients on opioids across three centres

Centre	Total No. patients	N. died	Person-time (years)	Crude incidence rate / 1,000 patients
	**Non-cancer**			
United Kingdom	993,294	32,409	1,864,384	17.4
United States (Boston)	43,243	401	84,425	4.7
Canada (Montréal)	26,116	401	46,544	8.6
	**Any pain**			
United Kingdom	453,601	8,908	861,227	10.3
United States (Boston)	16,699	217	32,432	6.6
Canada (Montréal)	13,363	169	24,067	7
	**Back, Lumbar or neck pain**			
United Kingdom	315,508	5,344	600,135	8.9
United States (Boston)	8,548	112	16,635	6.7
Canada (Montréal)	7,035	98	12,689	7.7

Supplementary Table 4: Fully adjusted models evaluating the association between opioid use and the risk of death: comparison of different follow up time for the 3 centres.

Centre / Opioid	24 months follow up		12 months follow up		6 months follow up	
	Hazard Ratio (95% CI)	*P*-Value	Hazard Ratio (95% CI)	*P*-Value	Hazard Ratio (95% CI)	*P*-Value
**United Kingdom**	**No. died=32,409**		**No. died=21,442**		**No. died=14,824**	
Codeine (reference)	Ref		Ref		Ref	
Dihydrocodeine	0.75 (0.69; 0.81)	< 0.001	0.73 (0.66; 0.80)	< 0.001	0.73 (0.65; 0.81)	0.73 (0.66;
Buprenorphine	5.74 (5.41; 6.10)	< 0.001	6.89 (6.45; 7.36)	< 0.001	8.25 (7.68; 8.86)	6.89 (6.45;
Hydrocodone						
Tramadol	1.08 (1.00; 1.17)	0.06	1.06 (0.96; 1.16)	0.25	1.12 (1.01; 1.25)	1.06 (0.96;
Hydromorphone	na		na		na	
Morphine	12.58 (11.87; 13.32)	< 0.001	14.36 (13.50; 15.29)	< 0.001	16.24 (15.18; 17.37)	14.36
Oxycodone	3.56 (2.94; 4.31)	< 0.001	3.70 (2.96; 4.63)	< 0.001	4.07 (3.16; 5.24)	3.70 (2.96;
Fentanyl	11.35 (10.69; 12.04)	< 0.001	13.74 (12.87; 14.66)	< 0.001	17.30 (16.13; 18.56)	13.74
Combination opioids	7.72 (7.30; 8.16)	< 0.001	8.48 (7.97; 9.02)	< 0.001	9.62 (8.99; 10.29)	8.48 (7.97;
Other opioids	0.86 (0.60; 1.22)	0.4	0.81 (0.54; 1.22)	0.31	0.90 (0.58; 1.40)	0.81 (0.54;
**United States (Boston)**	**No. died=401**		**No. died=303**		**No. died=232**	
Codeine (reference)	Ref		Ref		Ref	
Dihydrocodeine	na		na		na	
Buprenorphine	na		na		na	
Hydrocodone	1.06 (0.36; 3.15)	0.92	1.24 (0.39; 3.91)	0.71	1.22 (0.39; 3.84)	0.74
Tramadol	1.06 (0.42; 2.70)	0.90	1.26 (0.46; 3.44)	0.65	1.24 (0.45; 3.42)	0.67
Hydromorphone	1.58 (0.48; 5.19)	0.45	1.90 (0.55; 6.57)	0.31	1.50 (0.40; 5.62)	0.54
Morphine	8.62 (3.34; 22.27)	<.001	9.96 (3.57; 27.76)	< 0.001	9.47 (3.37; 26.62)	< 0.001
Oxycodone	1.43 (0.61; 3.32)	0.41	1.71 (0.68; 4.30)	0.25	1.72 (0.69; 4.32)	0.25
Fentanyl	dnc		dnc		dnc	
Combination opioids	1.49 (0.30; 7.44)	0.63	0.98 (0.11; 8.45)	0.99	1.16 (0.13; 9.99)	0.9
Other opioids	dnc		dnc		dnc	
**Canada (Montréal)**	**No. died=401**		**No. died=205**		**No. died=130**	
Codeine (reference)	Ref		Ref		Ref	
Dihydrocodeine	na		na		na	
Buprenorphine	na		na		na	
Hydrocodone	na		na		na	
Tramadol	na		na		na	
Hydromorphone	0.51 (0.05; 5.67)	0.59	dnc)		dnc	
Morphine	6.69 (1.35; 33.22)	0.02	9.21 (1.03; 82.58)	0.05	9.16 (1.02; 82.23)	0.05
Oxycodone	0.68 (0.06; 7.56)	0.76	1.26 (0.08; 20.20)	0.87	dnc	
Fentanyl	5.40 (1.12; 26.05)	0.04	7.53 (0.84; 67.59)	0.07	4.50 (0.41; 49.86)	0.22
Combination opioids	dnc		dnc		dnc	
Other opioids	dnc		dnc		dnc	

Abbreviations: dnc: category too small, did not converge, na: drug not available

Supplementary Table 5: Fully adjusted models evaluating the association between daily morphine milligram equivalents per day and the risk of death: comparison of different follow up time for the 3 centres.

Centre / Dose category	24 months follow up		12 months follow up		6 months follow up	
	Hazard Ratio (95% CI)	*P*-Value	Hazard Ratio (95% CI)	*P*-Value	Hazard Ratio (95% CI)	*P*-Value
**United Kingdom**	**No. died=32 409**		**No. died=21 442**		**No. died=14 824**	
< 50MME/day	Ref		Ref		Ref	
50 and <120 MME/day	5.33 (5.05; 5.63)	< 0.001	6.02 (5.67; 6.39)	< 0.001	6.79 (6.36; 7.24)	< 0.001
120 and < 200 MME/day	4.19 (3.80; 4.62)	< 0.001	4.93 (4.42; 5.50)	< 0.001	5.66 (5.02; 6.38)	< 0.001
>200 MME/day	4.66 (4.12; 5.28)	< 0.001	5.00 (4.34; 5.77)	< 0.001	5.49 (4.69; 6.43)	< 0.001
**United States (Boston)**	**No. died=401**		**No. died=303**		**No. died=232**	
< 50MME/day						
50 and <120 MME/day	1.86 (1.15; 3.01)	0.01	1.92 (1.18; 3.11)	0.008	1.81 (1.10; 2.96)	0.02
120 and < 200 MME/day	6.46 (2.95; 14.13)	< 0.001	5.64 (2.43; 13.10)	< 0.001	5.64 (2.42; 13.13)	< 0.001
>200 MME/day	11.52 (4.94; 26.86)	< 0.001	10.02 (3.98; 25.23)	< 0.001	10.09 (3.99; 25.53)	< 0.001
**Canada (Montréal)**	**No. died=401**		**No. died=205**		**No. died=130**	
< 50MME/day	Ref		Ref		Ref	
50 and <120 MME/day	0.87 (0.19; 3.91)	0.85	3.18 (0.53; 19.11)	0.21	2.57 (0.42; 15.55)	0.30
120 and < 200 MME/day	1.65 (0.21; 12.80)	0.63	dnc		dnc	
>200 MME/day	18.16 (4.99; 66.08)	< 0.001	12.76 (1.28; 127.69)	0.031	8.62 (0.79; 93.84)	0.08

Abbreviations: dnc: category too small, did not converge, na: drug not available

Supplementary Table 6: Number of patients using opioids and median number of days of use in the 2 years follow up by cohort for the United Kingdom.

Opioid and Concurrent Drug Use	All patients		Any Pain Patient		Back or Neck Pain	
	Any use N (%)	Median N days of use (std)	Any use N (%)	Median N days of use (std)	Any use N (%)	Median N days of use (std)
**Opioid**						
Codeine	763,752 (76.9%)	26.0 (29.0)	350,169 (77.2%)	26.0 (30.0)	241,913 (76.7%)	26.0 (29.0)
Dihydrocodeine	206,603 (20.8%)	23.0 (22.0)	97,707 (21.5%)	23.0 (22.0)	70,208 (22.3%)	23.0 (21.0)
Buprenorphine	12,380 (1.2%)	42.0 (118.0)	5,814 (1.3%)	42.0 (112.0)	4,073 (1.3%)	42.0 (108.0)
Hydrocodone						
Tramadol	155,443 (15.6%)	26.0 (41.0)	77,115 (17.0%)	26.0 (40.0)	55,585 (17.6%)	26.0 (40.0)
Hydromorphone						
Morphine	18,067 (1.8%)	21.0 (44.0)	7,507 (1.7%)	21.0 (51.0)	4,980 (1.6%)	21.0 (56.0)
Oxycodone	4,125 (0.4%)	30.0 (86.0)	2,014 (0.4%)	30.0 (82.0)	1,403 (0.4%)	30.0 (83.0)
Fentanyl	6,380 (0.6%)	32.0 (132.5)	2,510 (0.6%)	41.0 (144.0)	1,653 (0.5%)	42.0 (143.0)
Combination opioids	81,631 (8.2%)	17.0 (24.0)	40,592 (8.9%)	17.0 (22.0)	th29,849 (9.5%)	17.0 (22.0)
Other opioids	6,166 (0.6%)	24.0 (36.0)	2,840 (0.6%)	23.0 (31.5)	1,938 (0.6%)	23.5 (30.0)
**Concurrent drug**						
Antidepressant	265,193 (26.7%)	149.0 (375.0)	132,827 (29.3%)	140.0 (363.0)	89,283 (28.3%)	134.0 (351.0)
Antipsychotic	66,752 (6.7%)	26.0 (91.0)	30,475 (6.7%)	20.0 (52.0)	19,681 (6.2%)	21.0 (55.0)
Benzodiazepine	134,944 (13.6%)	12.0 (29.0)	74,983 (16.5%)	12.0 (23.0)	59,089 (18.7%)	12.0 (18.0)
Gabapentionoids	39,876 (4.0%)	88.0 (239.0)	22,575 (5.0%)	84.0 (223.0)	16,545 (5.2%)	81.0 (213.0)

Supplementary Table 7: Number of patients using opioids and median number of days of use in the 2 years follow up by cohort for Montreal.

Opioid and Concurrent Drug Use	All patients		Any Pain Patient		Back or Neck Pain	
	Any use N (%)	Median N days of use (std)	Any use N (%)	Median N days of use (std)	Any use N (%)	Median N days of use (std)
**Opioid**						
Codeine	15,299 (58.6)	6.0 (8.0)	7,505 (56.2)	7.0 (11.0)	3,862 (54.9)	7.0 (11.0)
Dihydrocodeine	Na	Na	Na	Na	Na	Na
Buprenorphine	Na	Na	Na	Na	Na	Na
Hydrocodone	Na	Na	Na	Na	Na	Na
Tramadol	Na	Na	Na	Na	Na	Na
Hydromorphone	7,159 (27.4)	7.0 (13.0)	4,025 (30.1)	8.0 (15.0)	2,197 (31.2)	9.0 (19.0)
Morphine	4,136 (15.8)	5.0 (7.0)	2,175 (16.3)	6.0 (11.0)	1,171 (16.6)	7.0 (11.0)
Oxycodone	5,167 (19.8)	7.0 (13.0)	2,999 (22.4)	9.0 (16.0)	1,606 (22.8)	9.0 (19.0)
Fentanyl	478 (1.8)	54.0 (154.0)	334 (2.5)	54.0 (163.0)	208 (3.0)	56.5 (191.5)
Combination opioids	1,633 (6.3)	6.0 (15.0)	1,005 (7.5)	7.0 (17.0)	591 (8.4)	9.0 (22.0)
Other opioids	492 (1.9)	6.0 (7.5)	279 (2.1)	6.0 (11.0)	138 (2.0)	6.0 (16.0)
**Concurrent drug**						
Antidepressant	16,639 (63.7)	305.0 (516.0)	8,640 (64.7)	316.0 (523.0)	4,467 (63.5)	291.0 (509.0)
Antipsychotic	4,195 (16.1)	281.0 (514.0)	1,915 (14.3)	278.0 (520.0)	1,050 (14.9)	279.0 (521.0)
Benzodiazepine	9,307 (35.6)	212.0 (496.0)	5,121 (38.3)	237.0 (502.0)	2,581 (36.7)	225.0 (489.0)
Gabapentionoids	4,700 (18.0)	142.0 (365.0)	3,239 (24.2)	144.0 (376.0)	1,891 (26.9)	143.0 (374.0)

Abbreviations : na: drug not available

Supplementary 8: Number of patients using opioids and median number of days of use in the 2 years follow up by cohort for the Boston.

Opioid and Concurrent Drug Use	All patients		Any Pain Patient		Back or Neck Pain	
	Any use N (%)	Median N days of use (std)	Any use N (%)	Median N days of use (std)	Any use N (%)	Median N days of use (std)
**Opioid**						
Codeine	3,638 (8.4)	26.0 (89.9)	1,382 (8.3)	30.0 (88.8)	754 (8.8)	32.0 (90.8)
Dihydrocodeine	na	na	na	na	na	na
Buprenorphine	na	na	na	na	na	na
Hydrocodone	4,946 (11.4)	27.0 (87.7)	1,918 (11.5)	30.0 (84.1)	1,017 (11.9)	28.0 (82.3)
Tramadol	4,725 (10.9)	42.0 (125.0)	2,330 (14.0)	38.0 (120.7)	1,214 (14.2)	36.0 (117.5)
Hydromorphone	2,027 (4.7)	16.0 (88.4)	889 (5.3)	18.0 (85.0)	429 (5.0)	22.0 (87.9)
Morphine	393 (0.9)	19.0 (92.9)	228 (1.4)	20.0 (92.2)	96 (1.1)	19.0 (77.6)
Oxycodone	29,015 (67.1)	19.0 (107.1)	10,740 (64.3)	21.0 (102.3)	5,412 (63.3)	21.0 (103.6)
Fentanyl	108 (0.2)	13.0 (94.2)	69 (0.4)	16.5 (104.0)	40 (0.5)	16.0 (98.6)
Combination opioids	1,030 (2.4)	8.0 (57.7)	544 (3.3)	7.0 (54.7)	247 (2.9)	5.0 (53.8)
Other opioids	59 (0.1)	32.0 (126.4)	33 (0.2)	32.0 (121.7)	13 (0.2)	32.0 (115.6)
**Concurrent drug**						
Antidepressant	13,932 (32.2)	66.0 (167.2)	6,621 (39.6)	58.0 (158.3)	3,149 (36.8)	56.0 (156.9)
Antipsychotic	2,887 (6.7)	46.0 (138.5)	1,494 (8.9)	40.0 (129.1)	713 (8.3)	36.0 (130.9)
Benzodiazepine	11,837 (27.4)	60.0 (162.1)	5,802 (34.7)	53.0 (154.9)	3,070 (35.9)	56.0 (156.7)
Gabapentionoids	6,110 (14.1)	49.0 (139.1)	3,679 (22.0)	48.0 (137.5)	1,790 (20.9)	49.0 (137.4)

Abbreviations : na: drug not available
